# Tubercular Consumption

**Published:** 1829

**Authors:** 


27. *Tubercular Consumption. Injurious effects of the antiphlogistic treatment.*—We have read with satisfaction, in the last number of the N. A. Med. and Surg. Jour. a paper by Dr. Parrish of Philadelphia, on the errors of the profession in the treatment of tubercular consumption. In the opinion of this able and experienced physician, it is a serious mistake to regard this malady as one of the phlegmasiæ, and treat it with bloodletting and other antiphlogistics. But let him speak for himself:

I do not believe that the condition of the lungs in tuberculous phthisis is at all analogous to ordinary inflammation, or that it is to be treated upon similar principles. The diseased action is peculiar, and requires peculiar modes of cure. It is true that genuine inflammation may, and often does, supervene upon the proper phthisical complaint, and tends to aggravate it. It is no uncommon circumstance for consumptive patients to be attacked with catarrh, and with acute pain in some part of the thorax. In this case, moderate depletion, either general or local, together with blisters to the breast, and other antiphlogistic measures, may be employed with advantage: but they should even here be used with caution, for fear of producing a degree of debility which the system in its diseased condition may not be able to surmount. But merely with the view of curing the tuberculous affection, bleeding should never be used: without diminishing the disorded action, it tends to enfeeble those vital energies which constitute the only defence against the progress of the invader.—278.

The object which the Doctor has in view, is to prevent the officious and exhausting interference of art in this incurable malady, rather than to point out new methods of treatment; he proceeds, however, to add his testimony to that of Sydenham, Rush and many other distinguished physicians, in favour of exercise. On this subject he holds the following language:

But, though the antiphlogistic course of treatment, and the use of powerful medicines, are calculated rather to co-operate with the disease of the lungs in reducing the system of the consumptive patient, than to relieve or eradicate the complaint, yet we are not therefore to surrender all hope, and yield up the sufferer an unresisting victim. On the contrary much may be done by exertion on the part of the individual affected in controlling the disease; and instances are not wanting in which it would seem to have been entirely conquered.

Vigorous exercise, and free exposure to the air, are by far the most efficient remedies in pulmonary consumption. It is not, however, that kind of exercise usually prescribed for invalids,—an occasional walk or ride in pleasant weather, with strict confinement in the intervals,—from which much good is to be expected. Daily and long continued riding on horseback, or in carriages over rough roads, is, perhaps, the best mode of exercise; but where this cannot be commanded, unremitting exertion of almost any kind in the open air, amounting even to labour, will be found highly beneficial. Nor should the weather be scrupulously studied. Though I would not advise a consumptive patient to expose himself recklessly to the severest inclemencies of the weather, I would nevertheless warn him against allowing the dread of taking cold to confine him on every occasion when the temperature may be low or the skies overcast.

I may be told that the patient is often too feeble to be able to bear exertion, but, except in the last stage, where every remedy must prove unavailing, I believe there are few who cannot use exercise without doors; and it sometimes happens, that they who are exceedingly debilitated find, upon making the trial, that their strength is increased by the effort, and that the more they exert themselves, the better able they are to support the exertion.

It is said by those who oppose this kind of treatment, that the lungs are in a state of inflammation, and therefore require rest. But admitting for a moment that the tubercles in pulmonary consumption are the result of ordinary inflammatory action of a chronic or subacute character,—an opinion, however, which I have already disclaimed,—yet the argument will not hold in the present case; for, from their very organization, the lungs cannot be at rest. From the moment we begin to breathe to the latest period of life, they are necessarily in continual motion. We cannot confine them as we can a diseased joint; and in attempting to restrain their motions by keeping the body at rest, without gaining our object, we generate a degree of irritability of system, which enables the local affection to operate upon the general health with a vast increase of deleterious effect.—279.

Dr. P. then goes on to narrate a number of cases in which the substitution of exercise for a course of depletion and polypharmacy, was attended with the happy effect of mitigating the sufferings and prolonging the life of the patient. How many of them are, in fact, examples of tubercular phthisis, we have no means of judging, as he has not given those symptoms which are distinctive of that disease from chronic bronchitis, and chronic pneumony with hepatization.

Our own observations coincide in the main, with those of our highly judicious friend, and hence we have been induced to present to our young and *energetic* readers, the results of his ample experience.

In a future essay he intends to give the fruits of his experience on the palliative pharmaceutic treatment of the same disease. For ourselves, we take this opportunity to say that on the whole we have found cupping, digitalis, colchicum, minute antimonials and opiates, of more efficacy in this respect than all other means. The great object seems to us to be, to moderate the wearing and ceaseless action of the heart, for to remove the tubercular irritation of the lungs which arouses their associate organ into preternatural contraction, is beyond the reach of the art, in its present imperfect state. Now, of the various means of fulfilling the indication here stated, we know of none so potent as the remedies just named. To be effective or even safe, they must, however, be administered with skill and caution. They may be given so sparingly as to do no good, or so freely as greatly to debilitate, if not destroy the patient. In their management, so as to moderate the frequency of the pulse, without impairing the powers of the system, consists the practical *tact* of the physician. If the *energy* of the circulation be great, the antimonial may be used more freely; if small, opium or one of the salts of morphia, should predominate.

In many cases we have seen the following formula (designed for our younger readers) produce the happiest effects:

℞. Thick mucilage of Gum Arabic, recently prepared, ℥iv.
Saturated tincture of Digitalis, or
Wine of Colchicum, ʒj.
Tartarized Antimony, Gr. j.
Sulphate of Morphia, Gr. j.
Mix.

A table spoonful, or half an ounce, may be taken from two to four times in the 24 hours.

In cases of great debility, the following palliative will be found to afford relief, and may also be recommended to the junior members of the profession:

℞. Gum. Arab. mucil. ℥iv.

Acid. Sulph. Aromat. ʒss.

Sulph Quin. Gr. viij.

Sulph. Morph. Gr. j.

Misce.

Against a salivation, prostrating doses of tartar emetic, extensive blistering, issues, and long applied tartar plasters, not less than copious blood letting, we feel it our duty to record an unqualified protest.

